# Dose‐dependent efficacy of a novel dsRNA‐based solution containing vadescana for *Varroa destructor* control

**DOI:** 10.1002/ps.71032

**Published:** 2026-06-29

**Authors:** Briana E Price, Ryan Kuesel, Taylor Reams, Brian Manley, James Masucci, Brandon K Hopkins

**Affiliations:** ^1^ Department of Entomology Washington State University Pullman WA USA; ^2^ GreenLight Biosciences, Research Triangle Park Durham NC USA

**Keywords:** biopesticide, RNA interference, *Varroa*, *Apis mellifera*, dose–response, vadescana

## Abstract

**BACKGROUND:**

A novel dsRNA‐based product, containing a molecule called vadescana, interferes with *Varroa destructor* (Anderson and Trueman) fecundity and shows promise as a new biopesticide strategy for *Varroa* management. This study defines optimal dosing rates for vadescana in the field by evaluating efficacy in full‐sized honey bee (*Apis mellifera* Linneas) hives to contribute essential preliminary information for vadescana development and implementation. Additionally, this study examined the impacts of various doses of vadescana on colony health by counting frames of bees and brood.

**RESULTS:**

Various concentrations, application volumes, and regimens (single or split application 3 weeks apart) of vadescana (2, 4, or 8 g/L) were applied to colonies and impacts were compared to colonies left untreated or treated with amitraz (positive control). Vadescana (4 g/L) applied at 4 g per brood box in a single dose was most effective at reducing *Varroa* populations for up to 18 weeks. Colony populations (average number of frames of bees and brood) remained unaffected among treatments over time.

**CONCLUSION:**

The results presented provide critical information for the development of vadescana by defining an optimal dose to use in full‐size honey bee colonies and supporting claims of insignificant honey bee colony‐level impacts. This study also introduces further research needs for broader applications and future deployment of this dsRNA‐based *Varroa* management option. © 2026 The Author(s). *Pest Management Science* published by John Wiley & Sons Ltd on behalf of Society of Chemical Industry.

## Graphical abstract

Vadescana is a novel dsRNA‐based product that targets *Varroa* fecundity. Vadescana applied at 4 g/brood box in one application was most effective at reducing *Varroa* populations for up to 18 weeks.

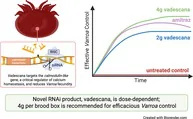

## 1 INTRODUCTION

Infestations by ectoparasitic mites, *Varroa destructor* (Anderson and Trueman), continue to be the main driver of honey bee (*Apis mellifera* Linnaeas) colony losses.^1^, ^2^, ^3^ By injuring via direct feeding on both adult and developing honey bees^4^, ^5^ and transmitting numerous honey bee‐related viruses,^6^, ^7^, ^8^, ^9^, ^10^, ^11^, ^12^ *Varroa* infestations are significantly detrimental to honey bee colony immunity and survival. The transmitted viruses can cause debilitating impacts such as trembling, malformed non‐functional wings, shortened abdomens, reduced lifespan, and inability to properly contribute to colony functions.^13^ As social insects, direct and indirect effects from *Varroa* infestations rapidly create immunological challenges at a colony level.^14^ In conjunction with viral infections, honey bees are exposed to a plethora of pesticides in various landscapes^15^, ^16^ and research has demonstrated that pesticide exposures can interrupt antiviral immune pathways, which weaken honey bees' ability to fight the viruses that *Varroa* vector.^17^ High *Varroa* infestations are also associated with lower success in queen introduction and mating,^18^ as well as issues with larval provisioning.^8^ This can lead to a cascade of other detrimental issues such as poor brood production and care, increased stress, and colony population decline. Since honey bees critically provide pollination services to hundreds of crops worldwide valued over $15 billion annually,^19^ there are continuous efforts to find efficacious and cost‐effective *Varroa* management strategies that are not harmful to honey bees.

A combination of control tactics is used as part of an integrated pest management (IPM) regimen for *Varroa* ^20^ control, including chemicals, or cultural controls such as drone brood removal,^21^ screened bottom boards,^22^ and breeding queens for *Varroa* resistance.^23^ Widespread adoption of comprehensive IPM protocols for *Varroa* is often limited, but according to Haber *et al*.^24^ commercial or hobbyist beekeepers often utilize only one miticidal active ingredient, such as amitraz‐based products or prefer only non‐chemical practices, respectively. The widespread and prolonged use of chemicals with the same active ingredients such as coumaphos, tau‐fluvalinate, or amitraz, and a high amount of inbreeding in *Varroa* populations^25^ has also led to the development of miticide resistance in *Varroa* populations, which diminishes treatment efficacy of these chemical options.^26^, ^27^, ^28^ Reasons for limited adoption of IPM in *Varroa* management include difficulty of implementing non‐chemical methods reliably due to the mite's complex biology, continued reliance on chemical controls despite resistance issues, and the cost associated with various cultural or mechanical management tactics.^20^ For example, beekeepers can create temporary breaks in the brood rearing cycle, forcing a larger proportion of mites into the dispersal phase, and increase a miticides efficacy^29^, ^30^, ^31^, ^32^, ^33^ but this may require additional time and labor for honey bees and beekeeping operations.

*Varroa* management strategies that have heavily relied on synthetic miticides with active ingredients coumaphos, tau‐fluvalinate, or amitraz, or organic compounds such as hop beta acids, formic acid, oxalic acid, or thymol face additional issues due to the developmental cycle of *Varroa* and the risk of toxic effects to developing and adult bees. Since *Varroas'* life cycle includes developing inside capped honey bee pupal cells, they have limited exposure to the miticides during early devolpment.^34^ Consequently, available miticide options vary in suitability and effectiveness during different honey bee population phases like when the colony is dormant with little to no brood or when the colony is increasing their population.^35^ While available synthetic compounds have been demonstrated to have negligible direct effects on bees,^36^ high concentrations of tau‐fluvalinate, coumaphos, or amitraz residues have been found in beeswax,^37^, ^38^ pollen,^39^ and honey stores^38^ within honey bee colonies; these chronic exposures may lead to issues such as increased brood mortality^39^ and genotoxic effects on bee immunity.^40^ The handful of organic compounds available for *Varroa* management also vary in effectiveness for *Varroa* control and impacts on honey bees. Hop beta acids have been demonstrated to have negligible toxicity to honey bees^41^ but products with this active ingredient are mostly ineffective during times of brood rearing.^42^ In some cases, oxalic acid or thymol have been demonstrated to have sublethal effects to honey bees; oxalic acid may cause decreased honey bee worker activity, nursing behavior, and longevity,^43^ or thymol may be harmful to honey bee brood and queens if applied during high outdoor temperatures.^44^ Formic acid can provide effective suppression of *Varroa* populations^45^ since it is the only option that can penetrate through the honey bee wax cappings,^46^, ^47^ but use of formic acid is limited by its temperature sensitivity. Additionally, formic acid can negatively impact honey bee memory^48^ and sometimes causes mortality of honey bee brood and queens.^49^, ^50^ Due to sub‐chronic exposures that impact bee health and widespread resistance development, there is a need for alternative, reliable control options for *Varroa* management.^51^

Researchers are working on developing biopesticides for *Varroa* management such as strains of entomopathogenic fungi that could persist in honey bee colonies, like *Metarhizium brunneum*.^52^ However, the use of RNA interference (RNAi) has emerged as a promising biopesticide for improving *Varroa* control by increasing *Varroa* mortality^53^, ^54^ and suppressing *Varroa* reproduction.^55^, ^56^, ^57^ RNAi involves the introduction of double‐stranded RNA (dsRNA), which is typically processed into small interfering RNAs (siRNAs). These siRNAs guide the RNA‐induced silencing complex (RISC) to target complementary mRNA sequences, leading to their degradation and subsequent suppression of protein expression.^58^

Vadescana is a novel dsRNA‐based option which initiates the RNAi mechanism targeting the *calmodulin* (GenBank accession number XM_022798728) and *calmodulin‐like* (GenBank accession number XM_022799184.1) genes in *Varroa*,^56^ critical regulators of calcium homeostasis.^59^ While vadescana has negligible direct lethality to *Varroa* adults, it does significantly reduce *Varroa* fecundity.^55^, ^56^, ^57^ Dose‐dependent effects of vadescana have been observed in field trials that spanned over 6 months,^60^ but optimal dosing remains unclear. The objective of this study focuses on evaluating possible dose–response of this novel dsRNA product and determining ideal dosing regimen(s) in full‐sized honey bee colonies. This study aims to define critical information needed to develop vadescana dosing for effective mite control for beekeepers and contribute essential information to the development of novel, sustainable strategies for *Varroa* management.

## 2 MATERIALS AND METHODS

A nested experimental design was employed during 2021–2022, with the same treatments and replications deployed across four states in the United States in 2021 and three states in the United States in 2022, utilizing colonies managed by four independent commercial beekeepers. In 2021, the experiment was initiated in the fall after honey harvest; 864 colonies (144 per treatment group) were used across four US states: Georgia, Florida, Louisiana, and North Carolina. In 2022, utilizing newly established colonies in May 2022, 540 (108 per treatment group) colonies across three states: California, Florida, and New York were used. Each apiary had 12 hives that were separately assigned to one of six treatments using a randomization tool. Within each state, three replicate locations separated by at least 8 km were chosen. Each location contained six apiaries, spaced more than 100 m apart but within a 2 km radius of each other. This spatial arrangement aimed to balance the need for similar environmental conditions within a location while minimizing the potential for drift and resultant mite migration. Colonies were arranged on migratory pallets of either three four‐way pallets or two six‐way pallets; all colonies on any one pallet received the same treatment as an additional buffer to potential confounding effects of drift. Due to the large scope of the trial, spanning multiple states and involving different beekeeper collaborators, a small cohort of investigators were assigned to collect all data parameters at the various locations; this minimized variability due to different personnel or human error.

**Figure 1:**
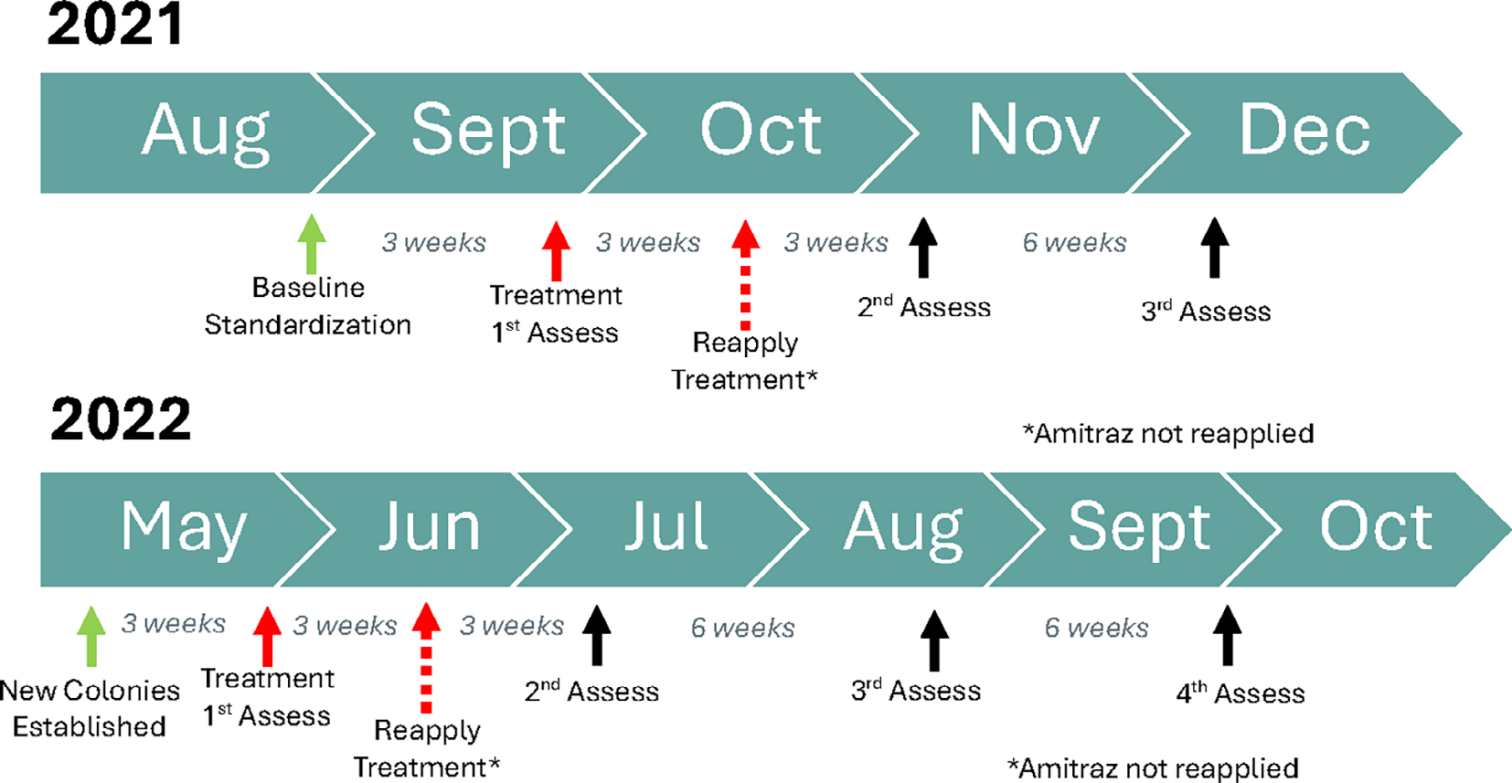
Approximate timeline and workflow of baseline assessment, treatment periods, and full assessments in 2021 and 2022. The timeline chart outlines the schedule of colony assessments and treatments in 2021 and 2022. Arrows mark key events such as baseline standardization, treatment applications, and assessments. Time intervals between events are noted.

All colonies started in single eight‐frame or 10‐frame Langstroth deep boxes (50.5 cm length × 36.3 cm width × 24.4 cm depth or 50.5 cm length × 41.3 cm width × 24.4 cm depth), but an additional brood box was added as needed. Colonies were standardized queen right (presence of a laying queen), had a consistent brood laying pattern, had no visible signs of disease based on visual inspection for common bee diseases, and contained six to eight frames of bees. Samples of approximately 300 adult bees were collected to estimate baseline *Varroa* populations; before field trials started, mite pressure was an average of 1.57 ± 0.12 and 1.81 ± 0.12 mites per 100 bees in 2021 and 2022, respectively. After baseline standardizations occurred, all colonies were assessed, labeled, and the appropriate treatment was applied to each colony. Three assessments were taken in 2021 and four were taken in 2022 (Fig. 1), with the first assessments happening immediately before colonies were treated.

### 2.1 Assessments

Assessments included quantifying colony population (frames of brood or adult bees),^61^, ^62^ indicating queen status (queen right, queenless, or a drone‐layer), and collecting samples of bees to quantify *Varroa* mite loads (mites per 100 bees). If colonies had less than two frames of bees or appeared or confirmed to be queenless during two subsequent inspections, they were marked dead.

#### 2.1.1 Mite counts in bee samples to quantify *Varroa* populations

During each assessment, bee samples were collected by shaking a frame of bees into a plastic collection bin and scooping approximately 300 bees into 133‐mL plastic sample cups with screw caps; samples were stored at −20 °C until analysis. All samples from a location during an assessment were weighed, then to approximate total number of bees collected for each location during that assessment, six subsamples of 100 bees per replication were separated and weighed (OHAUS Scout STX622 Portable Balance, Parsippany, NJ, USA) to determine the average weight per bee for each location at each assessment. To dislodge *Varroa*, sample cups were thawed, and contents were weighed. Sample cups were filled with soapy water and shaken at 300 osc/min for 15 min using a Burrell Wrist‐Action® shaker (Burrell Scientific LLC, Zelienople, PA, USA). Next, sample contents were poured through a #16 sieve to capture bees and a secondary #30 sieve containing lined with white nylon mesh to capture the dislodged mites. Bees were visually inspected for mites and samples were thoroughly washed to collect all mites in each sample. The summation of mites collected was divided by the total number of bees in each sample, which was estimated using the total weight of the bee sample divided by the average weight of a single bee as determined above to report mite loads as the standardized mites per 100 bees.^61^

#### 2.1.2 Colony populations

Frames of bees represent the number of frames covered greater than 75% with bees^61^ and was quantified using the California almond pollination method^62^ by tilting hive boxes and examining coverage of bees in the top and bottom, and counting seams with >75% coverage, and when colonies were in double‐deep colonies, taking the average of the two in each box. Frames of brood were determined by identifying both edges of the brood nest and counting the frames within the edges. Deep (10‐frame) and medium (eight‐frame) sized boxes were counted separately; counts of frames of bees or brood were standardized by dividing medium frames by 0.6, as the surface area of a medium frame is 60% of a deep frame.

### 2.2 Treatments

Vadescana (GreenLight Biosciences Inc., Medford, MA, USA), a dsRNA product that targets the *calmodulin* (XM_022798728) and *calmodulin‐like* (XM_022799184.1) genes of *Varroa*, was delivered to colonies utilizing permeable 500‐mL pouches containing various concentrations (0 = blank, 2, 4, or 8 g/L) of the active ingredient in a 61.6% weight/weight sucrose‐based solution with other proprietary ingredients (Table 1). Each pouch has small perforations on one side to allow the bees to extract the solution. The bees store the solution as they would for another nectar source and use it over time for brood food. Experiments included two controls: an untreated control with a ‘blank’ pouch containing the sucrose‐based solution only and Apivar® Varroa Mite Treatment Strips (active ingredient amitraz; Véto‐pharma, Palaiseau, France) with a blank pouch as a positive control. Blank sucrose pouches included all of the same proprietary ingredients as the treatment pouches except for the active dsRNA product. Apivar strips were applied according to the label. Three weeks later, during a second application, pouches were replaced with another containing the same concentration, a blank pouch, or nothing at all (Fig. 1 and Table 1).

**Table 1:** Description of treatments during both experimental years

	Treatment^†^ and formulation strength	First application description^‡^	Second application description^‡^	Total dsRNA per brood box over time (g)^§^
2 0 2 1	Blank untreated (− control)	One blank pouch	One blank pouch	0
	Amitraz (+ control)	Apivar strips and one blank pouch	One blank pouch	0
	Single vadescana 2 g/L	One pouch	One blank pouch	1
	Split vadescana 2 g/L	One pouch	One pouch	2
	Split vadescana 4 g/L	One pouch	One pouch	4
	Split vadescana 8 g/L	One pouch	One pouch	8
2 0 2 2	Blank untreated (− control)	One blank pouch	One blank pouch	0
	Amitraz (+ control)	Apivar strips and one blank pouch	One blank pouch	0
	Split vadescana 4 g/L	One pouch	One pouch	4
	Single vadescana Pair 2 g/L	Two pouches	None	2
	Single vadescana Pair 4 g/L	Two pouches	None	4

^†^ ‘Single’ refers to single application; ‘Split’ refers to reapplication 3 weeks after initial application; ‘Pair’ refers to two pouches given simultaneously.

^‡^ Each pouch contains 500 mL of 61.6% w/w sucrose‐based solution, +/− vadescana at 0 (‘blank’) 2, 4 or 8 g of active ingredient/L.

^§^ All colonies existed as single brood boxes at both application periods.

### 2.3 Statistical analysis

Data were analyzed in R version 4.4.3 (operating via RStudio, version 2024.12.1 + 563). Initial assessments in 2021 and 2022 were excluded from overall analyses because initial assessments took place immediately before treatments were applied. Instead, data from initial assessments were analyzed separately to ensure no bias was present between experimental groups at the start of the experiment.

#### 2.3.1 Mite counts in bee samples to quantify *Varroa* populations and colony populations

Cumulative mite loads were calculated and averaged by summing the number of mites per 100 bees across all assessments excluding assessment 1 (baseline) for each individual colony, then analyzed using restricted maximum likelihood linear mixed models (LMMs) after log+1 transformation with the function ‘lmer’ [R‐package ‘lme4’^63^]. State was included as a random effect to account for potential variability among states. Analyses for mite loads (mites per 100 bees), frames of bees, and frames of brood were performed across the two or three non‐initial assessments following methods for split‐block experiments^64^ using LMMs. Treatment, assessment, and the interaction treatment*assessment were included as fixed effects. State and apiary were included as random effects to account for potential variability among states and among apiary locations. Model fits were assessed visually with plots of model residuals and quantile–quantile plots to ensure adherence to model assumptions. Model selection was performed independently for each response variable. Potential analysis techniques were evaluated from least to most complex, starting with untransformed LMMs. All model assumptions of LMMs were met for frames of bees and frames of brood, so an LMM was selected without progressing through data transformation or generalized linear mixed models. Significant positive skew in mite loads was detected so further model techniques were explored. Square root transformation of mite loads did not allow the LMM to meet all assumptions. Log+1 transformation did allow the LMM to meet all model assumptions. An LMM with a log+1 transformed was selected to analyze mite loads without progressing to generalized linear mixed models. For each fitted model, aforementioned fixed effects were evaluated, and *P* values were calculated using the function ‘anova’ (R‐package ‘lmerTest’^65^). To examine the effects of treatment within individual assessments, a linear model was fitted for data within that assessment, and *P* values were calculated using the function ‘anova’ (R‐package ‘lmerTest’^65^). Post hoc pairwise contrasts were performed when the effect of ‘treatment’ was significant using the function ‘emmeans’ with a Sidak adjustment and *P* values for each pairwise comparison were calculated using the function ‘pairs’ (R‐package ‘emmeans’^66^).

#### 2.3.2 Colony mortality

Impact of treatment on colony mortality was examined in the last assessment only (assessment 3 in 2021 and assessment 4 in 2022) using a generalized linear mixed‐effects model with a binomial error distribution using in the function ‘glmer’ (R‐package ‘lme4’^63^). State and apiary were included as random effects to account for potential variability among states and among apiary locations.

## 3 RESULTS

### 3.1 *Varroa* populations

**Figure 2:**
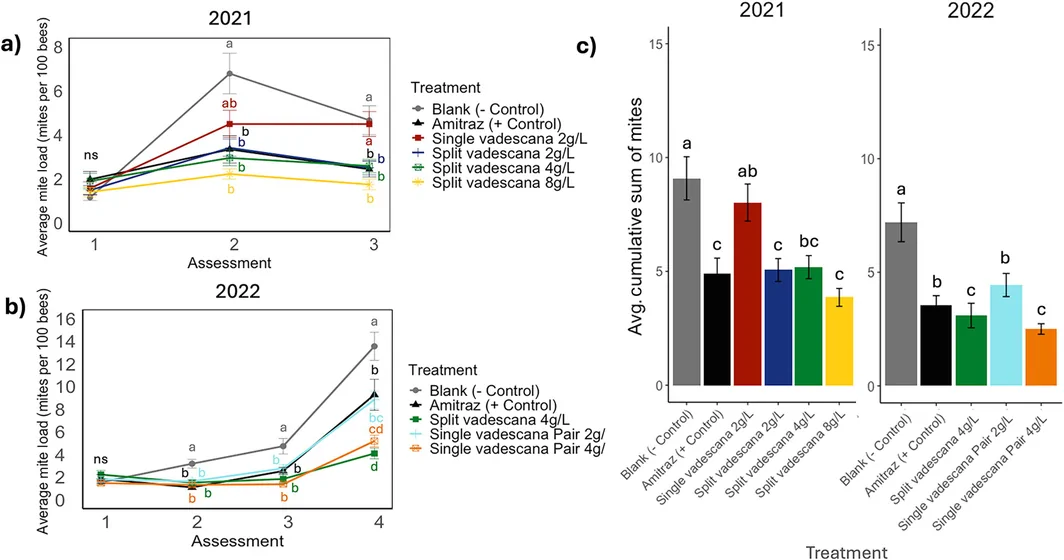
Average mite loads ± SE across three assessments during the 2021 field trial (a) and four assessments during the 2022 field trial (b). (a) and (b) show the average cumulative sum of mites ± SE per treatment group in 2021 and 2022. Assessment 1 was taken before treatment, Assessment 2 was 6 weeks post treatment, Assessment 3 was 12 weeks post treatment, and Assessment 4 was 18 weeks post treatment. Letters denote statistical differences among the treatment groups in each assessment according to pairwise contrasts with Tukey adjustment (*P < 0.05; ns, no significance, *P* > 0.05). (c) Bar chart showing data for various treatments in 2021 and 2022.

*Varroa* mite loads (mites per 100 bees) were different among treatment groups in both years (2021 *F*_5,786.51_ = 14.09, *P < 0.0001; 2022 *F*_4,482.41_ = 32.11, *P < 0.0001; Fig. 2) and assessments (2021 *F*_1,757.50_ = 11.46, *P < 0.0001; 2022 *F*_2,923.37_ = 650.13, *P < 0.0001). Treatment*assessment interactions varied by year, where no significant interaction was identified in 2021 (*F*_5,757.75_ = 1.45, *P =* 0.2015) but was identified in 2022 (*F*_8,923.77_ = 10.62, *P < 0.0001).

In 2021, mite loads in colonies treated with split applications of 4 g/L vadescana were similar to those in colonies treated with split applications of 8 g/L for up to 12 weeks (*P* = 0.2598), thus 4 g/L was chosen for a subsequent trial the following year. The single or split applications of 2 g/L vadescana performed similarly to the split application of 4 g/L in 2021 for up to 6 weeks (*P* = 0.3947, *P* = 0.9930, respectively), but after 6 weeks the single application of 2 g/L had similar mite loads as the untreated controls (*P* = 0.9998) and significantly higher mite loads than split applications of 2 and 4 g/L (*P = 0.0134, *P = 0.0176, respectively) (Fig. 2(a)). In 2022, the single application of 2 g/L applied as a pair of pouches did not work as effectively at lowering mite loads as split applications of 4 g/L (*P < 0.0001) or a single application of 4 g/L applied in a pair (*P = 0.0003) (Fig. 2(b)). Overall, colonies treated with split applications of 4 g/L vadescana had less than 2 mites per 100 bees 12–18 weeks after treatment during both field trials (Fig. 2(a),(b)), and a single application of 4 g/L vadescana applied as a pair of pouches kept *Varroa* mite loads under 4 mites per 100 bees for 18 weeks.

In 2021, colonies treated with amitraz or split applications of 2, 4, or 8 g/L of vadescana demonstrated significantly lower cumulative mite counts (average 3.9–5.2) than the blank controls (average 9.1) (*P = 0.0001, *P = 0.0013, *P = 0.0068, *P < 0.0001, respectively), while the single application (2 g/L) had similar cumulative mite counts (average 8.0) as the blank controls (*P* = 0.8627) (Fig. 2(c)). In 2021, amitraz‐treated colonies had similar mite loads as colonies that had split applications of 2, 4, or 8 g/L of vadescana (*P* = 0.9961, *P* = 0.9123, *P* = 0.5467, respectively), but colonies that got a single application (2 g/L) of vadescana exhibited significantly higher mites than amitraz‐treated colonies (*P = 0.0089; Fig. 2(c)).

In 2022, colonies treated with amitraz, split applications of 4 g/L of vadescana, or single applications of 2 or 4 g/L applied as a pair of pouches at one time had lower cumulative mite counts (average 2.5–4.4) than the blank controls (average 7.2) (*P = 0.0001, *P < 0.0001, *P = 0.0063, *P < 0.0001, respectively) (Fig. 2(c)). In 2022, amitraz‐treated colonies had significantly higher mite loads than colonies that had split applications of 4 g/L or a single application of 4 g/L vadescana applied as a pair of pouches (*P = 0.0078, *P = 0.0118, respectively), but had similar mite loads as colonies that got a single application (2 g/L) of vadescana (*P* = 0.8828; Fig. 2(c)).

### 3.2 Colony populations

**Figure 3:**
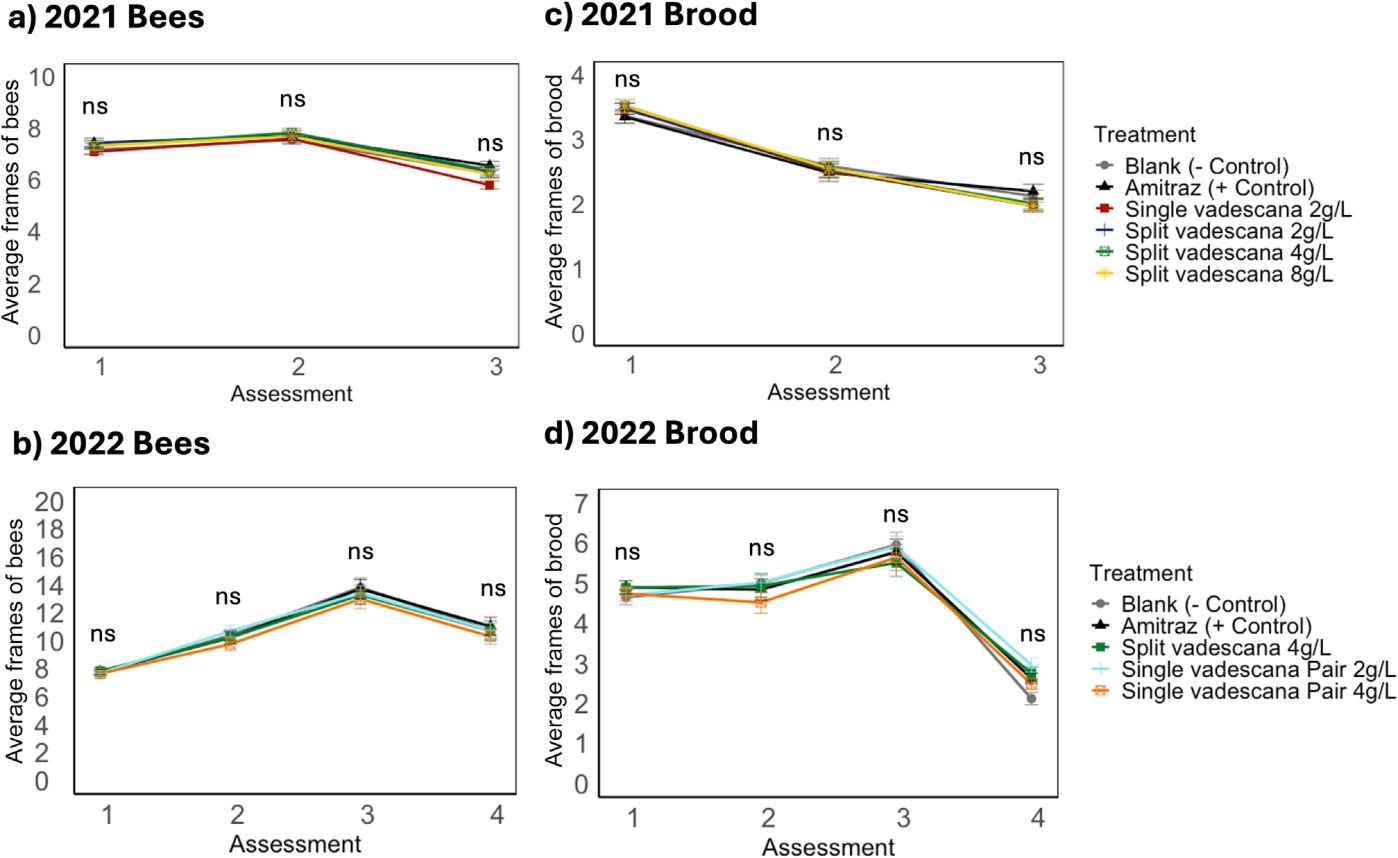
Average frames of bees (a) or brood (c) ± standard error (SE) observed in each assessment in the 2021 field trial and average frames of bees (b) or brood (d) ±SE observed in each assessment in the 2022 field trial. Assessment 1 was taken before treatment, Assessment 2 was 3–6 weeks post treatment, Assessment 3 was 9–12 weeks post treatment, and Assessment 4 was 15–18 weeks post treatment. ns indicates no significance according to pairwise contrasts with Tukey adjustment (*P* > 0.05). Assessments are plotted on the *x* axis and average frames on the *y* axis. Statistical significance is marked with letters (a, ab, b). ns, not significant.

There were no statistical differences in the number of frames of bees among treatment groups in 2021 (*F*_5,812.25_ = 1.37, *P* = 0.2318; Fig. 3(a)) or 2022 (*F*_4,491.07_ = 1.40, *P* = 0.2317; Fig. 3(b)) and no difference in the treatment*assessment interaction effect for frames of bees both years (2021: *F*_5,790.47_ = 1.16, *P* = 0.3296; 2022: *F*_8,942.39_ = 0.59, *P* = 0.7884). There were no statistical differences in the number of frames of brood among treatment groups in 2021 (*F*_5,839.70_ = 0.36, *P* = 0.8740; Fig. 3(c)) or 2022 (*F*_4,480.86_ = 0.71, *P* = 0.5829; Fig. 3(d)) and no difference in the treatment*assessment interaction effect for frames of brood both years (2021: *F*_5,803.03_ = 0.95, *P =* 0.4459; 2022: *F*_8,937.18_ = 1.32, *P =* 0.2288). Both years, there were significant ‘assessment’ effects for frames of bees (2021: F_1,790.78_ = 293.95, *P < 0.0001; 2022: *F*_2,942.06_ = 163.78, *P < 0.0001) and frames of brood (2021: *F*_1,803.48_ = 107.65, *P < 0.0001; 2022: *F*_2,937.40_ = 272.03, *P < 0.0001), which is expected since assessments were taken at various times of year and colony populations naturally fluctuate.

### 3.3 Colony mortality

**Figure 4:**
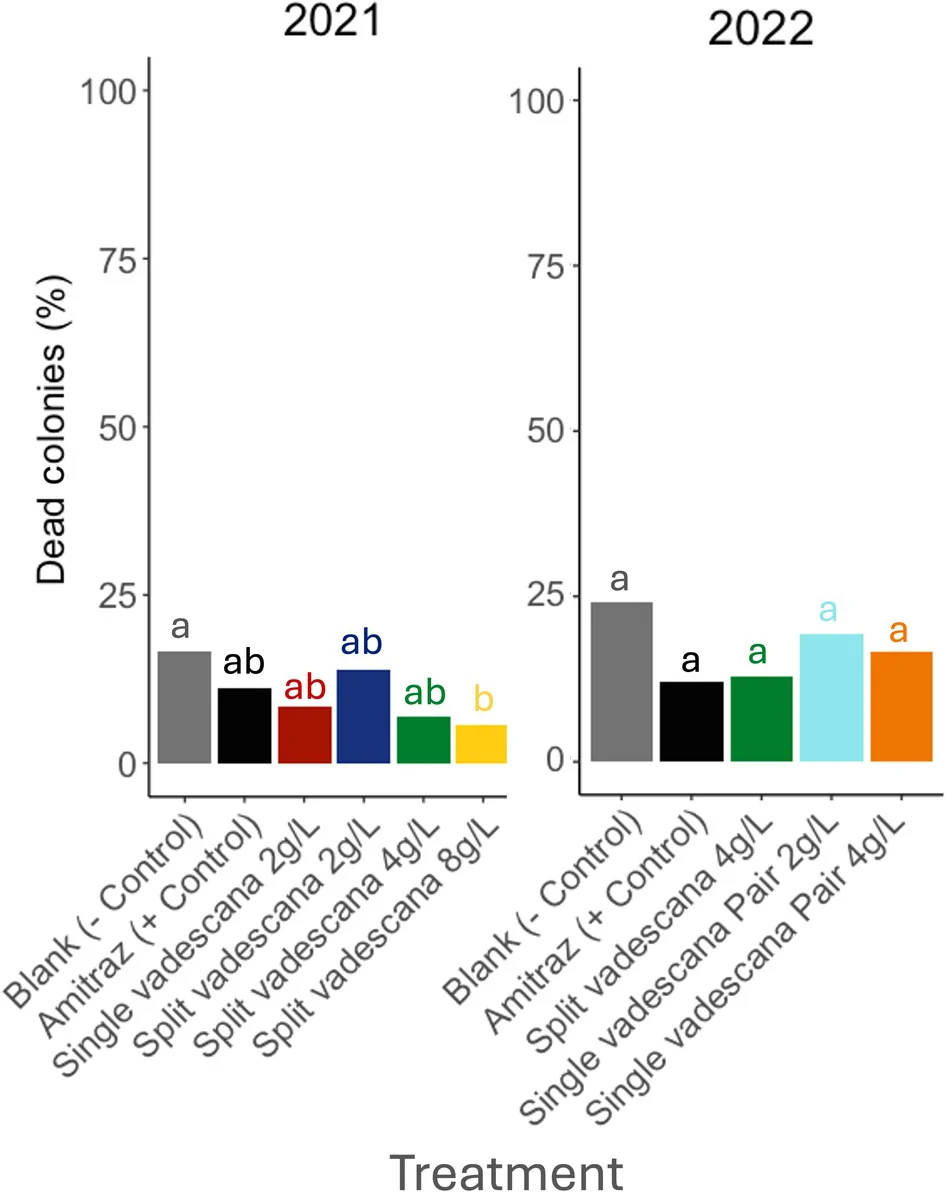
Percentage of dead colonies per treatment at the end of 2021 and 2022 assessments. Letters denote statistical differences among the treatment groups in each assessment according to pairwise contrasts with Tukey adjustment (*P < 0.05).

Colony mortality was statistically different across treatment groups in 2021 (*χ* ^2^ = 13.80, df = 5, *P = 0.0169), though all treatment groups experienced similar mortality as the untreated controls, ranging between ~7% and 17%, except colonies treated with 8 g/L of vadescana, which had significantly lower colony mortality than the untreated controls (~5%, *P = 0.0420; Fig. 4). In contrast, in 2022, colony mortality (~12–24%) was similar across all treatment groups (*χ* ^2^ = 7.77, df = 4, *P* = 0.0999; Fig. 4).

## 4 DISCUSSION

RNAi is an exciting new field of research, and its utilization opens new doors for the development of biopesticides for *Varroa destructor* control. As previously reported, *Varroa* are susceptible to ingested dsRNA.^53^, ^54^, ^55^ Studies have demonstrated an increase in adult *Varroa* mortality from the successful knockdown of vital *Varroa* genes.^53^, ^54^, ^56^ Further, three studies using vadescana reported a successful reduction in *Varroa* fecundity.^55^, ^56^, ^57^ Dose‐dependent effects of vadescana have been observed in field trials that spanned over six months^58^ but this study aimed to define an optimal dosing regimen to further develop vadescana as a novel strategy for *Varroa* management.

In McGruddy *et al*.,^55^ vadescana was delivered to honey bee nucleus (five‐frame) colonies via a sucrose‐based solution and researchers uncapped honey bee brood cells to observe mite populations 12 days post exposure; that study demonstrated that vadescana did not reduce the number of foundress mites, but did prevent the production of offspring. We have shown here that *Varroa* populations respond to vadescana in a dose‐dependent manner in colonies inside single Langstroth eight‐frame or 10‐frame boxes. In 2021, the single application of 2 g/L (1 g per brood box) was ineffective as it yielded similar mite loads compared to the untreated control during the experimental period. The 2 g/L dosage (2 g per brood box) applied in two application types (i.e. split application) or applied at once as a pair of pouches (i.e. single pair) significantly lowered mite loads for up to 12 weeks, although mite loads in colonies that were treated with a single application of 2 g/L increased 2‐fold alongside mite loads in amitraz‐treated colonies 18 weeks after treatment. Both years, the higher concentrations of vadescana (4 or 8 g/L) worked most effectively for the duration of the studies (up to 12 or 18 weeks, respectively); these application concentrations worked significantly better than the untreated controls and equally to amitraz 12 weeks after treatment. The 8 g/L treatment did not provide significantly greater *Varroa* control over 4 g/L, thus to balance efficacy and application cost, 4 g/L was used in the subsequent year's field trial. This plateau effect of RNAi dosage on the efficacy of *Varroa* control was unexpected, and further work should explore why such a plateau occurs.

In the 2022 field trial, vadescana was applied as a split application (one 500‐mL pouch of 4 g/L, 4 g per brood box) or single paired application (two 500‐mL pouches 2 or 4 g/L, 2 or 4 g per brood box) to investigate whether adding a larger quantity of vadescana would have extended efficacy and reduced the need for reapplication. In comparison, applications using 2 g/L did not work as effectively at lowering mite loads than 4 g/L applied as split applications or a single application of paired pouches. Colonies treated with split applications of 4 g/L vadescana had 1.5–3 mites per 100 bees 12–18 weeks after treatment during both field trials, and a single application of 4 g/L vadescana applied with a pair of pouches kept *Varroa* mite loads under 2 mites per 100 bees for 12 weeks, which is near the recommended treatment threshold for *Varroa*,^35^ but increased to ~4 mites per 100 bees around 18 weeks. Notably, these differences were seen without perfect control of *Varroa* drift between experimental colonies. Bees that drift from one colony to another often carry mature *Varroa* which enter the new colony and begin to reproduce. Preventing drift during field experiments is near impossible, but it is more likely to occur between nearby colonies.^67^, ^68^ We aimed to mitigate drift by assigning all colonies on any one pallet to receive the same treatment. Relative to treatment groups, drift should have occurred fairly randomly in our experiment, as the number of *Varroa* that drift from a colony is not significantly influenced by the mite load of the colony.^67^ Still, this mostly random drift could by chance reduce the true differences between treatment groups below what we measured, as one introduced *Varroa* can lead to exponentially more *Varroa* over an 18‐week duration due to their short maturation time. Overall, this study confirms that vadescana's efficacy is dose dependent and should be applied at 4 g/L (4 g per brood box) in a single or split application for future use. Our trials showed that a single application of 4 g/L was just as effective in reducing mite loads as a split application of the same concentration. Therefore, the single application method offers a successful approach to mite control without the need for reapplication.

Vadescana targets the *calmodulin* (XM_022798728) and *calmodulin‐like* (XM_022799184.1) genes in *Varroa*, which are critical regulators of calcium homeostasis needed for fertilization, egg activation, and other critical cellular functions in animals.^59^, ^69^ When a *Varroa* foundress enters a late‐stage honey bee larval cell, she must feed on honey bee larval hemolymph and receive stage‐specific volatile cues to stimulate egg production,^70^, ^71^ then lays eggs approximately 60 h after the honey bee brood cell is capped.^72^ Vadescana significantly lowers *Varroa* reproductive success of foundresses and viability of daughter mites and foundress mites recovered from cells displayed a white mass near their genital plates, which was indicative of unsuccessful reproduction.^55^, ^56^ *Varroa* spend their whole life cycle inside honey bee hives switching between dispersal and reproductive phases.^34^ During dispersal, adult mites cling to honey bees to find new brood cells^73^ or travel to new colonies^74^ and the reproductive phase involves entering honey bee brood cells to feed on developing brood while creating the next generation of mites.^75^ Since vadescana successfully reduces *Varroa* fecundity, it targets the reproductive phase of the *Varroa* life cycle, whereas all other current miticide options are limited to targeting the *Varroa* dispersal phase only. Targeting the reproductive phase is an advantageous new approach since reproductive *Varroa* can evade most miticides when they are protected under the wax layer of honey bee pupal cells.^34^ While not addressed in this study, vadescana's mode of action could have the potential for controlling another ectoparasitic mite of honey bees, such as *Tropilaelaps* spp. *Tropilaelaps* spp. also feed on honey bee brood and vector diseases,^76^ but unlike *Varroa*,^77^ feeding on developing honey bees is not needed for *Tropilaelaps* spp. reproductive maturation or oviposition^78^ so they are considered more prolific than *Varroa*. According to Bulgarella *et al*.,^79^ one species of *Tropilaelaps* that is an emerging threat to global beekeeping,^80^ *T. mercedesae*, has the potential to be susceptible to vadescana based on siRNA sequence matching^79^ Future research investigating the utilization and effectiveness of vadescana or other dsRNAs for controlling *Tropilaelaps* spp. populations should be explored.

If used for *Varroa* control, honey bees would undoubtedly be exposed to vadescana. Calmodulin genes are highly conserved in animals,^59^ so risk to honey bees is being thoroughly examined^55^, ^81^; dsRNA sequences can be designed to be species‐specific and minimize risk to non‐target organisms.^82^ Vadescana has been designed to be nucleotide‐sequence‐specific to *Varroa* calmodulin messenger RNA (mRNA). This high specificity to *Varroa* was confirmed through bioinformatic analyses of potential gene silencing effects of 37 non‐target arthropod genomes, including several species of honey bees.^79^ While McGruddy *et al*.^55^ reported no detriment to honey bee pupal survival, and Merk *et al*.^81^ reported positive effects of vadescana on foraging activity compared to control colonies, this study examined colony population (frames of brood or adult bees) since it is a standardized predictor of future colony success.^83^ We observed similar colony populations across all treatments, indicating that vadescana did not cause any measurable detriment to honey bee brood development or adult viability. All treatment groups, excluding 8 g/L vadescana‐treated colonies, experienced similar mortality as the controls, ranging between ~7% and 17% in 2021 and ~12–24% in 2022, and colonies treated with 8 g/L had lower mortality (~5%). While mite loads were significantly decreased in vadescana‐treated colonies, similar colony mortality among treatment groups could be driven by a multitude of external factors such as locality, management differences between beekeeping operation, nutrition, or other factors that can cause the death of a colony beyond *Varroa* infestation. While we did not differentiate between colonies that died due to *Varroa*‐induced colony collapse and those that died due to those potential operational failures, we are confident there were very few incidental mortality events across all trials, meaning the vast majority of colony losses were related to declining health of the colony. To capture significant impacts on mortality caused by *Varroa* infestation levels, the study would have needed to follow colonies for much longer (~1 year or longer) and record the reason for colony death, since the long‐term effects of *Varroa* infestations are not always observed within an 18‐week timeframe. While not explored in this study, possible effects of vadescana on honey bee queen reproduction and mating quality should be explored since sperm viability in queens can be negatively impacted by in‐hive *Varroa* management chemicals currently used by beekeepers.^84^, ^85^

There is a high demand for new efficacious *Varroa* management tools, including dsRNA‐based solutions and other biopesticides, since current chemical treatments are losing effectiveness due to resistance development, can cause sublethal impacts to honey bees, and may leave harmful residues in hive products. Vadescana offers a precise, targeted RNAi approach that can disrupt *Varroa* fecundity and two products with vadescana as active ingredients were recently registered for *Varroa* management by the US Environmental Protection Agency. Now that an optimal dose of vadescana in full‐sized brood boxes has been defined, it should be tested in multi‐year field studies in rotation with other chemical options in an IPM approach. To fully characterize appropriate timing of applications, the efficacious dose (4 g per brood box) described here should be tested during times of different honey bee population seasonal phases and in rotation with other control options. Lastly, once effective IPM strategies are investigated in full‐scale field trials, proper outreach and education about how RNAi works in a *Varroa* IPM system will likely encourage adoption from beekeepers.^86^, ^87^ This study contributes critical insights for development of vadescana as an dsRNA‐based *Varroa* management tool by defining an optimal dose, confirming no honey bee colony‐level impacts, and highlighting future research needs for broader applications in IPM.

## 5 CONCLUSION

This study demonstrates dose‐dependent efficacy of vadescana for *Varroa* control in full sized (eight‐frame or 10‐frame) honey bee brood boxes and confirms the safety of vadescana to honey bee colony populations. Doses of 4 or 8 g/L (4 or 8 g/brood box) were most effective at reducing *Varroa* populations, and therefore 4 g/L was chosen as the preferred concentration to include in subsequent field testing and was submitted for regulatory approval to the US Environmental Protection Agency. The recommended dose, based on this work, is 1 L of product per brood box, which delivers 4 g vadescana per brood box (i.e. 4 g/L). Colony population remained unaffected by treatment including a 2× dose (8 g/L). Colony mortality was similar across all treatments, except colonies treated with 8 g/L had slightly less mortality on average, further confirming the safety of vadescana to honey bee colonies. This study contributes to a larger case of prefatory information needed for the development and deployment of vadescana for RNAi‐based *Varroa* management. Multi‐year full‐scale field trials are underway to investigate vadescana in an IPM system across several seasons and geographic regions.

## CONFLICT OF INTEREST

Brian Manley and James D. Masucci are employed by GreenLight Biosciences Inc., the developer of vadescana. The other authors declare no conflict of interest.

## Data Availability

The data that support the findings of this study are available from the corresponding author upon reasonable request.

## References

[1] Guzmán‐Novoa E , Eccles L , Calvete Y , Mcgowan J , Kelly PG and Correa‐Benítez A , *Varroa destructor* is the main culprit for the death and reduced populations of overwintered honey bee (*Apis mellifera*) colonies in Ontario, Canada. Apidologie 41:443–450 (2010).

[2] Brodschneider R , Gray A , Adjlane N , Ballis A , Brusbardis V , Charrière JD *et al*., Multi‐country loss rates of honey bee colonies during winter 2016/2017 from the COLOSS survey. J Apicultural Res 57:452–457 (2018).

[3] Steinhauer N , Kulhanek K , Antúnez K , Human H , Chantawannakul P and Chauzat MP , Drivers of colony losses. Curr Opin Insect Sci 26:142–148 (2018). 29764654 10.1016/j.cois.2018.02.004

[4] Kanbar G and Engels WJ , Ultrastructure and bacterial infection of wounds in honey bee (*Apis mellifera*) pupae punctured by *Varroa* mites. Parasitol Res 90:349–354 (2003). 12684884 10.1007/s00436-003-0827-4

[5] Ramsey SD , Ochoa R , Bauchan G , Gulbronson C , Mowery JD , Cohen A *et al*., *Varroa destructor* feeds primarily on honey bee fat body tissue and not hemolymph. Proc Natl Acad Sci USA 116:1792–1801 (2019). 30647116 10.1073/pnas.1818371116 PMC6358713

[6] Chen Y , Pettis JS , Evans JD , Kramer M and Feldlaufer MF , Transmission of Kashmir bee virus by the ectoparasitic mite *Varroa destructor* . Apidologie 35:441–448 (2004).

[7] Yue C and Genersch E , RT‐PCR analysis of deformed wing virus in honeybees (*Apis mellifera*) and mites (*Varroa destructor*). J Gen Virol 86:3419–3424 (2005). 16298989 10.1099/vir.0.81401-0

[8] Aronstein KA , Saldivar E , Vega R , Westmiller S and Douglas AE , How *Varroa* parasitism affects the immunological and nutritional status of the honey bee, Apis mellifera. Insects 3:601–615 (2012). 26466617 10.3390/insects3030601 PMC4553578

[9] Mondet F , de Miranda JR , Kretzschmar A , Le Conte Y and Mercer AR , On the front line: quantitative virus dynamics in honeybee (*Apis mellifera* L.) colonies along a new expansion front of the parasite *Varroa destructor* . PLoS Pathog 10:e1004323 (2014). 25144447 10.1371/journal.ppat.1004323 PMC4140857

[10] Piou V , Schurr F , Dubois E and Vétillard A , Transmission of deformed wing virus between *Varroa destructor* foundresses, mite offspring and infested honey bees. Parasit Vectors 15:333 (2022). 36151583 10.1186/s13071-022-05463-9 PMC9502634

[11] Damayo JE , McKee RC , Buchmann G , Norton AM , Ashe A and Remnant EJ , Virus replication in the honey bee parasite, *Varroa destructor* . J Virol 97:e01149‐23 (2023). 37966226 10.1128/jvi.01149-23 PMC10746231

[12] Molinatto G , Mondet F , Marzachi C , Alaux C , Bassi E , Dievart V *et al*., Seasonal variations of the five main honey bee viruses in a three‐year longitudinal survey. Apidologie 56:23 (2025).

[13] de Miranda JR , Gauthier L , Ribiere M and Chen YP , Honey bee viruses and their effect on bee and colony health, in Honey Bee Colony Health: Challenges and Sustainable Solutions, ed. by Sammataro D and Yoder JA . CRC Press, Boca Raton, FL. pp. 71–102 (2011).

[14] Fefferman NH , Traniello JFA , Rosengaus RB and Calleri DV , Disease prevention and resistance in social insects: modeling the survival consequences of immunity, hygienic behavior, and colony organization. Behav Ecol Sociobiol 61:565–577 (2007).

[15] Traynor KS , Tosi S , Rennich K , Steinhauer N , Forsgren E , Rose R *et al*., Pesticides in honey bee colonies: establishing a baseline for real world exposure over seven years in the USA. Environ Pollut 279:116566 (2021). 33839524 10.1016/j.envpol.2021.116566

[16] Zhang G , Kuesel R , Olsson R , Reed R , Liu X and Hopkins B , Pesticide exposure patterns in honey bees during migratory pollination. J Hazard Mater 480:135910 (2024). 39321480 10.1016/j.jhazmat.2024.135910

[17] Harwood GP and Dolezal AG , Pesticide–virus interactions in honey bees: challenges and opportunities for understanding drivers of bee declines. Viruses 12:566 (2020). 32455815 10.3390/v12050566 PMC7291294

[18] Rateb SH , Abdel‐Rahman MF and Sanad RE , The relationship between *Varroa destructor* infestation and virgin queen's acceptance, mating success and onset of oviposition in honeybee colonies. Egypt Acad J Biol Sci 3:207–212 (2010).

[19] Chopra SS , Bakshi BR and Khanna V , Economic dependence of US industrial sectors on animal‐mediated pollination service. Environ Sci Tech 49:14441–14451 (2015). 10.1021/acs.est.5b03788 26575436

[20] Jack CJ and Ellis JD , Integrated pest management control of *Varroa destructor* (Acari: Varroidae), the Most damaging Pest of (*Apis mellifera* L. (hymenoptera: Apidae)) colonies. J Insect Sci 21:6 (2021). 10.1093/jisesa/ieab058 PMC8449538 34536080

[21] Calderone NW , Evaluation of drone brood removal for management of *Varroa destructor* (Acari: Varroidae) in colonies of *Apis mellifera* (Hymenoptera: Apidae) in the northeastern United States. J Econ Entomol 98:645–650 (2005). 16022287 10.1603/0022-0493-98.3.645

[22] Delaplane KS , Berry JA , Skinner JA , Parkman JP and Hood WM , Integrated pest management against *Varroa destructor* reduces colony mite levels and delays treatment threshold. J Apicultural Res 44:157–162 (2005).

[23] Guichard M , Dietemann V , Neuditschko M and Dainat B , Advances and perspectives in selecting resistance traits against the parasitic mite *Varroa destructor* in honey bees. Genet Selection Evol 52:71 (2020). 10.1186/s12711-020-00591-1 PMC7694340 33246402

[24] Haber AI , Steinhauer NA and vanEngelsdorp D , Use of chemical and nonchemical methods for the control of *Varroa destructor* (Acari: Varroidae) and associated winter Colony losses in US beekeeping operations. J Econ Entomol 112:1509–1525 (2019). 31008501 10.1093/jee/toz088

[25] Beaurepaire AL , Krieger KJ and Moritz RFA , Seasonal cycle of inbreeding and recombination of the parasitic mite *Varroa destructor* in honey bee colonies and its implications for the selection of acaricide resistance. Infect Genet Evol 50:49–54 (2017). 28216419 10.1016/j.meegid.2017.02.011

[26] Hernández‐Rodríguez CS , Marín Ó , Calatayud F , Mahiques MJ , Mompó A , Segura I *et al*., Large‐scale monitoring of resistance to coumaphos, amitraz, and pyrethroids in *Varroa destructor* . Insects 12:27 (2021). 33406622 10.3390/insects12010027 PMC7824307

[27] Millán‐Leiva A , Marín Ó , Christmon K , Vanengelsdorp D and González‐Cabrera J , Mutations associated with pyrethroid resistance in *Varroa* mite, a parasite of honey bees, are widespread across the United States. Pest Manag Sci 77:3241–3249 (2021). 33728766 10.1002/ps.6366

[28] Bahreini R , González‐Cabrera J , Hernández‐Rodríguez CS , Moreno‐Martí S , Muirhead S , Labuschagne RB *et al*., Arising amitraz and pyrethroids resistance mutations in the ectoparasitic *Varroa destructor* mite in Canada. Sci Rep 15:1587 (2025). 39794392 10.1038/s41598-025-85279-6 PMC11724071

[29] Giacomelli A , Pietropaoli M , Carvelli A , Iacoponi F and Formato G , Combination of thymol treatment (Apiguard®) and caging the queen technique to fight *Varroa destructor* . Apidologie 47:606–616 (2016).

[30] Jack CJ , Van Santen E and Ellis JD , Evaluating the efficacy of oxalic acid vaporization and brood interruption in controlling the honey bee pest *Varroa destructor* (Acari: Varroidae). J Econ Entomol 113:582–588 (2020). 31909423 10.1093/jee/toz358

[31] Berry JA , Braman SK , Delaplane KS and Bartlett LJ , Inducing a summer brood break increases the efficacy of oxalic acid vaporization for *Varroa destructor* (Mesostigmata: Varroidae) control in *Apis Mellifera* (hymenoptera: Apidae) Colonies. J Insect Sci 23:14 (2023). 10.1093/jisesa/iead085 PMC10699866 38055946

[32] Gabel M , Scheiner R and Büchler R , Immediate and long‐term effects of induced brood interruptions on the reproductive success of *Varroa destructor* . Apidologie 54:20 (2023).

[33] Aurell D , Bruckner D , Steury TD and Williams GR , Treating newly split *Apis Mellifera* honey bee colonies with organic miticides—an opportunity for integrated pest management of *Varroa destructor* mites (Mesostigmata: Varroidae). J Econ Entomol, 118:1495–1503 (2025). 40591393 10.1093/jee/toaf126 PMC12412293

[34] Rosenkranz P , Aumeier P and Ziegelmann B , Biology and control of *Varroa destructor* . J Invertebr Pathol 103:S96–S119 (2010). 19909970 10.1016/j.jip.2009.07.016

[35] Honey Bee Health Coalition , Tools for Varroa management: a guide to effective Varroa sampling & control, 8th edn. Keystone Policy Center, Keystone (CO) (2022).

[36] Dai P , Jack CJ , Mortensen AN , Bustamante TA and Ellis JD , Chronic toxicity of amitraz, coumaphos and fluvalinate to *Apis mellifera* L. larvae reared *in vitro* . Sci Rep 8:5635 (2018). 29618776 10.1038/s41598-018-24045-3 PMC5884784

[37] Mullin CA , Frazier M , Frazier JL , Ashcraft S , Simonds R , vanEngelsdorp D *et al*., High levels of miticides and agrochemicals in north American apiaries: implications for honey bee health. PLoS One 5:e9754 (2010). 20333298 10.1371/journal.pone.0009754 PMC2841636

[38] Kast C , Droz B and Kilchenmann V , Toxicity of coumaphos residues in beeswax foundation to the honey bee brood. Environ Toxicol Chem 42:1816–1822 (2023). 37144826 10.1002/etc.5645

[39] Sanchez‐Bayo F and Goka K , Pesticide residues and bees – a risk assessment. PLoS One 9:e94482 (2014). 24718419 10.1371/journal.pone.0094482 PMC3981812

[40] Sabová L , Maruščáková IC , Koleničová S , Mudroňová D , Holečková B , Sabo R *et al*., The adverse effects of synthetic acaricide tau‐fluvalinate (tech.) on winter adult honey bees. Environ Toxicol Pharmacol 92:103861 (2022). 35398274 10.1016/j.etap.2022.103861

[41] Rademacher E , Harz M and Schneider S , The development of HopGuard® as a winter treatment against *Varroa destructor* in colonies of *Apis mellifera* . Apidologie 46:748–759 (2015).

[42] DeGrandi‐Hoffman G , Ahumada F , Probasco G and Schantz L , The effects of beta acids from hops (*Humulus lupulus*) on mortality of *Varroa destructor* (Acari: Varroidae). Exp Appl Acarol 58:407–421 (2012). 22767150 10.1007/s10493-012-9593-2 PMC3487002

[43] Schneider S , Eisenhardt D and Rademacher E , Sublethal effects of oxalic acid on *Apis mellifera* (Hymenoptera: Apidae): changes in behaviour and longevity. Apidologie 43:218–225 (2012).

[44] Floris I , Satta A , Cabras P , Garau VL and Angioni A , Comparison between two thymol formulations in the control of *Varroa destructor*: effectiveness, persistence, and residues. J Econ Entomol 97:187–191 (2004). 15154435 10.1093/jee/97.2.187

[45] Jack CJ , Boncristiani H , Prouty C , Schmehl DR and Ellis JD , Evaluating the seasonal efficacy of commonly used chemical treatments on *Varroa destructor* (Mesostigmata: Varroidae) population resurgence in honey bee colonies. J Insect Sci 24:11 (2024). 10.1093/jisesa/ieae011 PMC11132127 38805652

[46] Emsen B and Dodoloğlu A , Efficacy of different organic compounds against bee mite (*Varroa destructor* Anderson and Trueman) in honey bee (*Apis mellifera* L.) colonies. J Anim Vet Adv 10:802–805 (2011).

[47] Kubásek J , Svobodová K , Půta F and Krejčí AB , Honeybees control the gas permeability of brood and honey cappings. iScience 25:105445 (2022). 36388978 10.1016/j.isci.2022.105445 PMC9650039

[48] Gashout HA , Guzman‐Novoa E , Goodwin PH and Correa‐Benítez A , Impact of sublethal exposure to synthetic and natural acaricides on honey bee (*Apis mellifera*) memory and expression of genes related to memory. J Insect Physiol 121:10401 (2020). 10.1016/j.jinsphys.2020.104014 31923391

[49] Elzen PJ , Westervelt D and Lucas R , Formic acid treatment for control of *Varroa destructor* (Mesostigmata: Varroidae) and safety to *Apis mellifera* (Hymenoptera: Apidae) under southern United States conditions. J Econ Entomol 97:1509–1512 (2004). 15568336 10.1603/0022-0493-97.5.1509

[50] Giovenazzo P and Dubreuil P , Evaluation of spring organic treatments against *Varroa destructor* (Acari: Varroidae) in honey bee *Apis mellifera* (hymenoptera: Apidae) colonies in eastern Canada. Exp Appl Acarol 55:65–76 (2011). 21442305 10.1007/s10493-011-9447-3

[51] Dietemann V , Pflugfelder J , Anderson D , Charrière JD , Chejanovsky N , Dainat B *et al*., *Varroa destructor*: research avenues towards sustainable control. J Apicultural Res 51:125–132 (2012).

[52] Han JO , Naeger NL , Hopkins BK , Sumerlin D , Stamets PE , Carris LM *et al*., Directed evolution of metarhizium fungus improves its biocontrol efficacy against *Varroa* mites in honey bee colonies. Sci Rep 11:10582 (2021). 34011994 10.1038/s41598-021-89811-2 PMC8134475

[53] Garbian Y , Maori E , Kalev H , Shafir S and Sela I , Bidirectional transfer of RNAi between honey bee and *Varroa destructor*: *Varroa* gene silencing reduces *Varroa* population. PLoS Pathol 8:e1003035 (2012). 10.1371/journal.ppat.1003035 PMC3534371 23308063

[54] Muntaabski I , Scannapieco AC , Liendo MC , Niz JM , Russo R and Salvador R , Bacterially expressed dsRNA induces *Varroa destructor* gene knockdown by honey bee‐mediated oral administration. J Apicultural Res 61:511–518 (2022).

[55] McGruddy RA , Smeele ZE , Manley B , Masucci JD , Haywood J and Lester PJ , RNA interference as a next‐generation control method for suppressing *Varroa destructor* reproduction in honey bee (*Apis mellifera*) hives. Pest Manag Sci 80:4770–4778 (2024). 38801186 10.1002/ps.8193

[56] Smeele ZE , McGruddy RA , Baty JW , Manley B , Narva K , Neef ED *et al*., An RNA interference biopesticide reduces reproduction of the honey bee parasite *Varroa destructor* by down‐regulating embryo development pathways. Pest Manag Sci 82:2694–2707 (2026). 41346097 10.1002/ps.70406 PMC12886170

[57] Rawn D , Prouty C , Gautam A , Jamison M , Talton W , Youngs K *et al*., Evaluating the new product Norroa™ against *Varroa destructor* in managed honey bee (*Apis mellifera*) colonies. Front Insect Sci 6:1751606 (2026). 41890275 10.3389/finsc.2026.1751606 PMC13013518

[58] Almeida R and Allshire RC , RNA silencing and genome regulation. Trends Cell Biol 15:251–258 (2005). 15866029 10.1016/j.tcb.2005.03.006

[59] Clapham DE , Calcium signaling. Cell 131:1047–1058 (2007). 18083096 10.1016/j.cell.2007.11.028

[60] Masucci J , Developing double‐stranded RNA as a new *Varroa* control product. Am Bee J 160:1–6 (2020).

[61] Dietemann V , Nazzi F , Martin SJ , Anderson DL , Locke B , Delaplane KS *et al*., Standard methods for *Varroa* research. J Apic Res 52:1–54 (2013).

[62] Goodrich BK and Goodhue RE , Are all colonies created equal? The role of honey bee colony strength in almond pollination contracts. Ecol Econ 177:106744 (2020).

[63] Bates D , Mächler M , Bolker B and Walker S , Fitting linear mixed‐effects models using lme4. J Stat Softw 67:1–48 (2015).

[64] Piepho H‐P , Edmondson R and Yaseen M , AgriTutorial – Tutorial analysis of some agricultural experiments. AgriTutorial vignette (2019).

[65] Kuznetsova A , Brockhoff PB and Christensen RHB , lmertest. R package version 3.1–3 (2020).

[66] Lenth R , Singmann H , Love J , Buerkner P and Herve M , Emmeans – Estimated Marginal Means, aka Least‐Squares Means R package version 1.8.4–1 (2019).

[67] Forfert N , Natsopoulou ME , Frey E , Rosenkranz P , Paxton RJ and Moritz RF , Parasites and pathogens of the honeybee (*Apis mellifera*) and their influence on inter‐colonial transmission. PLoS One 10:e0140337 (2015). 26451849 10.1371/journal.pone.0140337 PMC4599887

[68] Seeley TD and Smith ML , Crowding honeybee colonies in apiaries can increase their vulnerability to the deadly ectoparasite *Varroa destructor* . Apidologie 46:716–727 (2015).

[69] Stricker SA , Comparative biology of calcium signaling during fertilization and egg activation in animals. Dev Biol 211:157–176 (1999). 10395780 10.1006/dbio.1999.9340

[70] Garrido C and Rosenkranz P , The reproductive program of female *Varroa destructor* mites is triggered by its host, Apis mellifera. Exp Appl Acarol 31:269–273 (2003). 14974691 10.1023/b:appa.0000010386.10686.9f

[71] Frey E , Odemer R , Blum T and Rosenkranz P , Activation and interruption of the reproduction of *Varroa destructor* is triggered by host signals (*Apis mellifera*). J Invertebr Pathol 113:56–62 (2013). 23376006 10.1016/j.jip.2013.01.007

[72] Ifantidis MD , Ontogenesis of the mite *Varroa jacobsoni* in worker and drone honey‐bee brood cells. J Apicultural Res 22:200–206 (1983).

[73] Xie X , Huang Z and Zeng Z , Why do *Varroa* mites prefer nurse bees? Sci Rep 6:28228 (2016). 27302644 10.1038/srep28228 PMC4908398

[74] DeGrandi‐Hoffman G , Ahumada F , Zazueta V , Chambers M , Hidalgo G and DeJong EW , Population growth of *Varroa destructor* (Acari: Varroidae) in honey bee colonies is affected by the number of foragers with mites. Exp Appl Acarol 69:21–34 (2016). 26910522 10.1007/s10493-016-0022-9 PMC4824817

[75] Lin Z , Qin Y , Page P , Wang S , Li L , Wen Z *et al*., Reproduction of parasitic mites *Varroa destructor* in original and new honeybee hosts. Ecol Evol 8:2135–2145 (2018). 29468031 10.1002/ece3.3802 PMC5817142

[76] de Guzman LI , Williams GR , Khongphinbunjong K and Chantawannakul P , Ecology, life history, and Management of Tropilaelaps Mites. J Econ Entomol 110:319–332 (2017). 28334185 10.1093/jee/tow304

[77] Reams T and Rangel J , Understanding the enemy: a review of the genetics, behavior and chemical ecology of *Varroa destructor*, the parasitic mite of *Apis mellifera* . J Insect Sci 22:18 (2022). 10.1093/jisesa/ieab101 PMC8825774 35137134

[78] Woyke J , *Tropilaelaps clareae* females can survive for 4 weeks when given open bee brood of *Apis mellifera* . J Apic Res 33:21–25 (1994).

[79] Bulgarella M , Reason A , Baty JW , McGruddy RA , Gordon ERL , Devisetty UK *et al*., *In silico* analysis of potential off‐target effects of a next‐generation dsRNA acaricide for Varroa mites (*Varroa destructor*) and lack of effect on a bee‐associated arthropod. Insects 16:317 (2025). 40266823 10.3390/insects16030317 PMC11942661

[80] Chantawannakul P , Ramsey S , vanEngelsdorp D , Khongphinitbunjong K and Phokasem P , Tropilaelaps mite: an emerging threat to European honey bee. Curr Opin Insect Sci 26:69–75 (2018). 29764663 10.1016/j.cois.2018.01.012

[81] Merk J , Anastasi M , McGruddy RA , Manley B , Felden A and Lester PJ , Longevity and foraging performance of honey bees treated with an RNAi‐based *Varroa destructor* biopesticide. Sci Rep 16:8208 (2026). 41786813 10.1038/s41598-026-38557-w PMC12963394

[82] Whyard S , Singh AD and Wong S , Ingested double‐stranded RNAs can act as species‐specific insecticides. Insect Biochem Mol Biol 39:824–832 (2009). 19815067 10.1016/j.ibmb.2009.09.007

[83] Delaplane KS , van der Steen J and Guzman‐Novoa E , Standard methods for estimating strength parameters of *Apis mellifera* colonies. J Apicultural Res 52:1–12 (2013).

[84] Burley LM , Fell RD and Saacke RG , Survival of honey bee (Hymenoptera: Apidae) spermatozoa incubated at room temperature from drones exposed to Miticides. J Econ Entomol 101:1081–1087 (2008). 18767713 10.1603/0022-0493(2008)101[1081:sohbha]2.0.co;2

[85] Rangel J and Tarpy DR , In‐hive miticides and their effect on queen supersedure and colony growth in the honey bee (*Apis mellifera*). J Environ Anal Toxicol 6:3 (2016).

[86] McGruddy R , Haywood J and Lester PJ , Beekeepers support the use of RNA interference (RNAi) to control *Varroa destructor* . Insects 15:539 (2024). 39057271 10.3390/insects15070539 PMC11276693

[87] Lester P , Bulgarella M , Manley B , Masucci J , McGruddy R , Merk J *et al*., Norroa™ as a dsRNA biopesticide for *Varroa destructor*: insights from New Zealand research on mode of action, field efficacy, non‐target effects, and social acceptance. Front Insect Sci 6:1814622 (2026). 42344312 10.3389/finsc.2026.1814622 PMC13286927

